# Carbon nanotubes on nanoporous alumina: from surface mats to conformal pore filling

**DOI:** 10.1186/1556-276X-9-390

**Published:** 2014-08-12

**Authors:** Jinghua Fang, Igor Levchenko, Zhao Jun Han, Samuel Yick, Kostya Ken Ostrikov

**Affiliations:** 1 Plasma Nanoscience Laboratories, Manufacturing Flagship, CSIRO, P.O. Box 218, Lindfield, NSW 2070, Australia; 2 School of Physics, University of Melbourne, Parkville, VIC 3010, Australia; 3 Complex Systems, School of Physics, The University of Sydney, Sydney, NSW 2006, Australia; 4 Institute for Future Environments and School of Chemistry, Physics, and Mechanical Engineering, Queensland University of Technology, Brisbane, QLD 4000, Australia

**Keywords:** Carbon nanotubes, Alumina template, Plasma treatment

## Abstract

Control over nucleation and growth of multi-walled carbon nanotubes in the nanochannels of porous alumina membranes by several combinations of posttreatments, namely exposing the membrane top surface to atmospheric plasma jet and application of standard S1813 photoresist as an additional carbon precursor, is demonstrated. The nanotubes grown after plasma treatment nucleated inside the channels and did not form fibrous mats on the surface. Thus, the nanotube growth mode can be controlled by surface treatment and application of additional precursor, and complex nanotube-based structures can be produced for various applications. A plausible mechanism of nanotube nucleation and growth in the channels is proposed, based on the estimated depth of ion flux penetration into the channels.

**PACS:**

63.22.Np Layered systems; 68. Surfaces and interfaces; Thin films and nanosystems (structure and non-electronic properties); 81.07.-b Nanoscale materials and structures: fabrication and characterization

## Background

Hybrid structures based on nanowires and nanotubes grown on solid matrices are promising materials for various applications ranging from nanoelectronics [1,2] and biotechnology [3] to superhydrophobic surfaces [4], reinforced composite materials [5] and polymers [6]. Application of the hybrid nanotube-based structures for water desalination can have alluring prospects [7,8]. Among others, nanoporous aluminium oxide (alumina) membranes are often used as a base for such structures [9,10]. Carbon nanotubes embedded in the nanoporous alumina membrane demonstrate promising properties [11], but controllability of the nanotube growth in the membrane is still a challenge. Carbon nanotubes and graphene flakes have been successfully grown using high-temperature reactions in the gas phase [12,13]. However, this method has not been able to synthesize nanotube arrays and meshes with controlled structure and morphology. In particular, it is still a challenge to grow carbon nanotubes selectively in the channels only or on the membrane surface. Moreover, the mechanisms of nanotube nucleation and growth in channels and on the featured membrane surface are still far from being completely understood.

One possible way to enhance the controllability and outcome of the growth process and to fabricate sophisticatedly designed nanotube-based complex nanomaterials is to involve additional treatment methods, such as plasma-based processing [14]. Atmospheric-pressure plasma jets [15,16], microwave [17,18], magnetron [19] and RF-based systems [20] are the common setups used for the plasma-enhanced nanofabrication. The atmospheric-pressure plasma jets and inductively coupled plasmas were particularly useful for the fabrication of one- and two-dimensional carbon-based nanostructures such as self-organized carbon connections [21] and graphene flakes [22]. In the plasma- or hit gas-based growth processes, the precursors containing carbon (such as acetylene, methane, ethanol vapour or other similar gases) dissociate to molecular, atomic and ion species [23], then deposit onto the catalyst nanoparticles and nucleate on the catalyst surface. The further growth of carbon nanomaterials (graphene flakes, carbon nanowires or nanotubes) is sustained by the incorporation of carbon atoms via bulk and surface diffusion. The presence of ion and electron fluxes in the material flow to the substrate surface intensifies the surface-based growth processes and results in the formation of unique structures [24,25].

In this paper, we demonstrate that by involving (i) plasma posttreatment of the nanoporous alumina membranes and (ii) additional carbon precursor (photoresist), one can control the morphology of the nanotube array grown on the membrane. Moreover, (iii) a plausible mechanism of the nanotube nucleation and growth in the channels is proposed based on the estimated depth of ion flux penetration into the channels. Our experiments show that denser arrays of nanotubes can be formed due to the plasma treatment, and importantly, the upper surface of the membrane can be kept free of nanotubes confined inside the membrane channels.

## Methods

**Figure 1:**
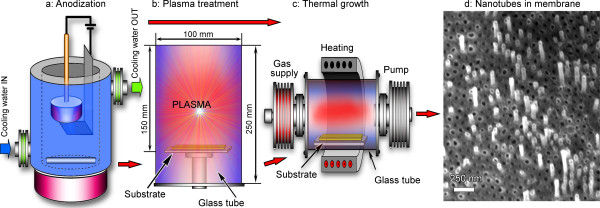
**Schematic of the process.** After anodization in oxalic acid **(a)**, the samples are subject to plasma pretreatment **(b)** or directly supplied to the thermal furnace for carbon nanotube growth **(c)**. SEM image **(d)** shows the carbon nanotubes partially embedded in the nanoporous alumina membrane.

Schematic of the plasma-assisted fabrication process is shown in Figure 1. The process starts from electrochemical fabrication of free-standing (i.e. not attached to other substrates) alumina membrane using a two-step anodization in an electrochemical anodization cell by the voltage reductional sequence process [26]. The nanoporous membranes with an average pore diameter of about 20 to 50 nm and external diameter of about 15 mm were produced from a thin (250 μm) high-purity (99.999%) aluminium foil. The anodization voltage was regulated in a range of 20 to 40 V to control the pore size, so the lower voltage produced smaller pores. The process was conducted in oxalic (0.4 M) acid solution used as electrolyte at temperature 0°C, controlled using the cooling system LAUDA Alpha RA8 (Thomas Scientific, Swedesboro, NJ, USA). The ready nanoporous membrane had a thickness of at least 20 to 25 μm, and thus, the as-grown free-standing samples could be handled using tweezers. Morphologically, the membranes are thin transparent films pierced with straight channels through the entire depth. A scheme of the electrochemical anodization cell is shown in Figure 1a. More details of this process and properties of the nanoporous alumina membranes can be found elsewhere [27].

The further experimental study was organized as follows. Firstly, all samples were divided into the three series, each series consisting of three samples for the nanotube growth in CH_4_, C_2_H_4_ and C_2_H_2_ precursor gases (see Table 1). The samples of the first series were coated with a 0.5-nm-thick Fe layer (series ‘Fe only’). Next, all samples of the second series were spin-coated with S1813 photoresist (propylene glycol monomethyl ether acetate, molecular weight 132.16, which contains 55% of carbon according to the linear formula CH_3_CO_2_CH(CH_3_)CH_2_OCH_3_,) and then coated with a 0.5-nm-thick Fe layer (series ‘Fe + S1813’). Finally, all samples of series 3 (series ‘Fe + S1813 + Plasma’) were loaded into a vacuum chamber of the inductively coupled plasma reactor (Figure 1b). The chamber (glass tube with the diameter of 100 mm and the length of 250 mm) was evacuated to the pressure lower than 10^−6^ Torr, and Ar was then injected to reach the pressure of 3 × 10^−2^ Torr. Afterwards, the radio-frequency power (50 W, 13.56 MHz) was applied, and alumina templates were treated by the discharge plasma for 5 min. During treatment, the samples were installed on Si wafers insulated from the supporting table. Hence, the top surfaces of the alumina membranes were under floating potential (about 15 to 20 V in this case), and the ion current to the surface was compensated with electron current from the plasma. No external heating was used. After the plasma treatment, the samples were spin-coated with S1813 photoresist and then coated with a 0.5-nm-thick Fe layer. Such a thin layer cannot form a continuous film at elevated temperatures. During the process, it fragments and forms an array of nanosized islands [28]. Scanning electron microscope (SEM) images of the catalyst layer fragmented after heating can be found elsewhere [29].

**Table 1:** Conditions and results of experiments

**Series**	**Process ( *T *, °C)**	**Carbon precursor**	**Result**
Fe only	900	CH_4_	No CNT
	750	C_2_H_4_	CNT on top only
	700	C_2_H_2_	CNT on top only, curved, amorphous
Fe + S1813	900	CH_4_	CNT in channels and top
	750	C_2_H_4_	CNT in channels and top
	700	C_2_H_2_	CNT in channels and top
Fe + S1813 + Plasma	900	CH_4_	CNT in channels
	750	C_2_H_4_	CNT in channels
	700	C_2_H_2_	CNT in channels

The growth temperatures were optimized to produce specific outcomes. CNT, carbon nanotube.

**Figure 2:**
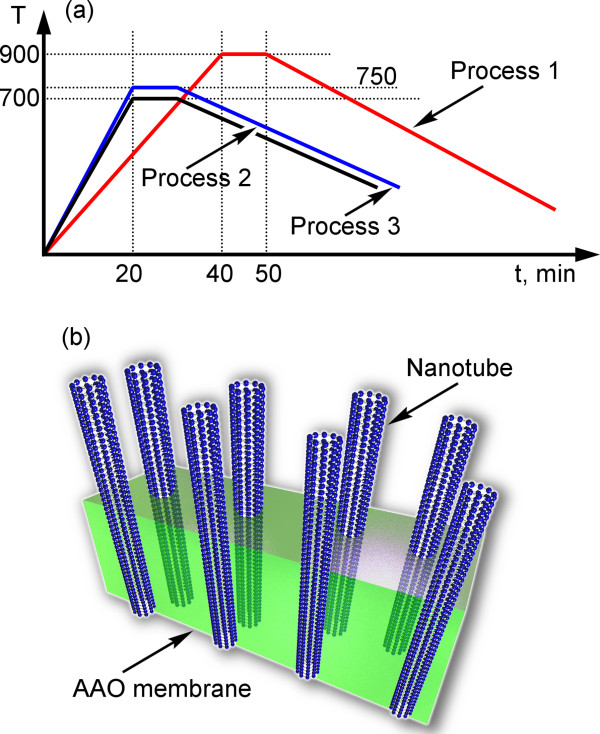
**Temperature/time dependencies and three-dimensional visualization of the targeted structure. (a)** Temperature/time dependencies for three processes used for growing carbon nanotubes on alumina membranes. **(b)** Three-dimensional visualization of the targeted structure - carbon nanotubes partially embedded in the nanoporous alumina matrix (membrane).

The growth of carbon nanotubes on the as-prepared nanoporous membranes was conducted as follows. First, three prepared samples (one sample from the Fe only series, one sample from the Fe + S1813 series and one sample from the Fe + S1813 + Plasma series) were loaded into the thermal furnace, and the growth process was conducted for 10 min at 900°C in a CH_4_ + H_2_ + Ar gas mixture at atmospheric pressure after 40-min-long heating. A gas supply system (bottles and mass flow controllers) was used to maintain the desired flow rates (up to 1,000 sccm for He or Ar) in the reaction area (quarts tube). After the growth, the samples were cooled down slowly, together with the furnace. Next, other three prepared samples (one from each series) were loaded into the thermal furnace, and the carbon nanotube growth was conducted for 10 min at 750°C in a C_2_H_4_ + H_2_ + Ar gas mixture at atmospheric pressure. Finally, three samples from each series were treated for 10 min at 700°C in C_2_H_2_ + H_2_ + Ar. Note that all the samples were coated with Fe which is an efficient catalyst for carbon nanotube growth due to the high carbon solubility in Fe and ability to form iron carbides [30]. The process sequence diagrams for all the samples are shown in Figure 2a, and the three-dimensional representation of one of the targeted structure (carbon nanotubes in the nanoporous membrane) is shown in Figure 2b. The process was repeated on several samples to confirm the reproducibility. With the process conditions kept constant, no significant variation in the results (nanotube size, system morphology, etc.) were found on the samples that have undergone the same process.

The ready samples were then examined using field-emission scanning electron microscope (FE-SEM, type Zeiss Auriga, Carl Zeiss, Inc., Oberkochen, Germany) operated at electron beam energy of 1 to 5 keV with an InLens secondary electron detector. The structure of the nanotubes was studied by transmission electron microscopy (TEM) technique using a JOEL 2100 microscope (JEOL Ltd., Akishima-shi, Japan) operated at the electron beam energy of 200 keV. Micro-Raman spectroscopy was performed using a Renishaw inVia spectrometer (Renishaw PLC, Wotton-under-Edge, UK) with laser excitations of 514 and 633 nm and a spot size of approximately 1 μm^2^. Raman spectra from multiple spots were collected to perform the statistical analysis of the samples.

## Results and discussion

**Figure 3:**
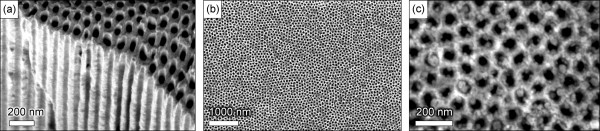
**SEM images. (a)** SEM image of the nanoporous alumina membrane (side and top view) before the nanotube growth. The membrane is formed by densely packed, highly ordered channels. **(b, c)** Low- and high-resolution SEM images of the membrane (top view) after the treatment by ‘900°C’ process, Fe only series, see Table 1. Only slight carbonous contaminations can be noted on the top of the membrane.

The results of the above described experiments are summarized in Table 1, in line with the process reagents and temperatures. SEM image of the typical nanotube array grown in the nanoporous membrane is shown in Figure 1d. The temperature-time dependencies for all three processes used for growing carbon nanotubes on alumina membranes and the three-dimensional visualization of the targeted structure (carbon nanotubes partially embedded in the nanoporous alumina membrane) are shown in Figure 2.A representative SEM image of the alumina membrane prepared for the nanotube growth is shown in Figure 3a. From this image, one can see that the membrane is formed with straight, long, open channels arranged into the regular network. The samples from Fe only series (only Fe layer on the top of the nanoporous membrane) do not exhibit carbon nanotubes on the top of membrane or inside the channels. Only slight traces of carbonous contaminations sometimes blocking the channels can be found on the membrane (Figure 3b,c shows low- and high-resolution images of the samples, top views).

**Figure 4:**
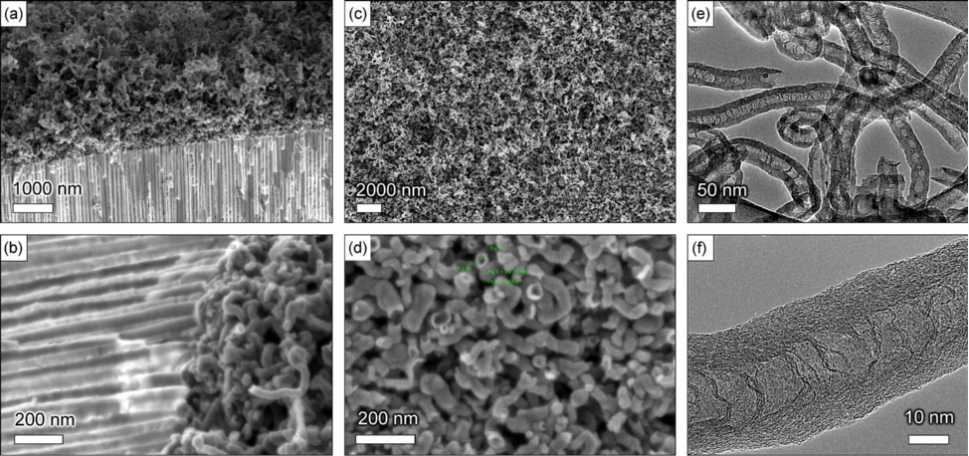
**SEM and TEM images of carbon nanotubes grown in 750°C process, Fe only series (C**_**2**_**H**_**4**_**, no S1813; Table** 1**). (a, b)** Side view, nanotubes are present on the membrane top only, the channels are empty; **(c, d)** top view; and **(e, f)** the multi-walled nanotubes contain approximately 25 to 30 walls.

Figure 4 shows SEM and TEM images of the carbon nanotubes grown in 750°C process, Fe only series (C_2_H_4_, no S1813, see Table 1). Figure 4a,b shows the cross-sectional side views of the alumina membrane (the cross-sectional side views were prepared by notching the membrane surface followed by careful cleavage through the whole depth, as well as by partial cutting using the focused ion beam on the scanning electron microscope), demonstrating the ‘empty’ channels which do not contain any nanotubes and a dense fibrous mat of curved, entangled carbon nanotubes on the top of membrane. Thorough examination of the channels to the whole membrane thickness using SEM has revealed that the channels are empty through their entire length, i.e. over the entire membrane thickness. One more sectional side view with the empty channels is shown in Additional file 1: Figure S1. The diameter of a typical nanotube is 40 to 50 nm. These nanotubes most likely nucleated on the iron nanoislands formed on the top of the membrane [31].Figure 4c,d shows SEM images of the top surfaces of respective samples. A dense fibrous mat of thick carbon nanotubes covers the top surface, and nanopores of the alumina membrane are completely clogged. Interestingly, as one can notice in Figure 4d, some nanotubes are open. The total thickness of the carbon nanotube mat can be estimated from SEM images and reaches several micrometres.To better characterize the grown nanotubes, high-resolution TEM (HRTEM) technique was used. Figure 4e shows the TEM image of the nanotubes found on the membrane top. Some nanotubes are open, and no metal catalyst particles were found on TEM images. Moreover, one can conclude from this image that the nanotubes are multi-walled with clear inside channels containing two-dimensional graphene-like flakes that bridge the innermost walls. The high-resolution TEM image shown in Figure 4f confirms these finding. The nanotube walls have a thickness of about 10 nm and consist of 25 to 30 graphitic layers. The crystalline structure is rather good, with most of the graphitic layers aligned along the nanotube axis.

Similar experiments on the growth of nanotubes in C_2_H_2_ atmosphere without S1813 have shown quite similar results (curved nanotubes on the alumina membrane top, no nanotubes in the membrane channels), but the TEM analysis has revealed a nearly amorphous structure. This observation is likely due to the rather low process temperature which was not sufficient for crystallization, even in the presence of Fe catalyst.

**Figure 5:**
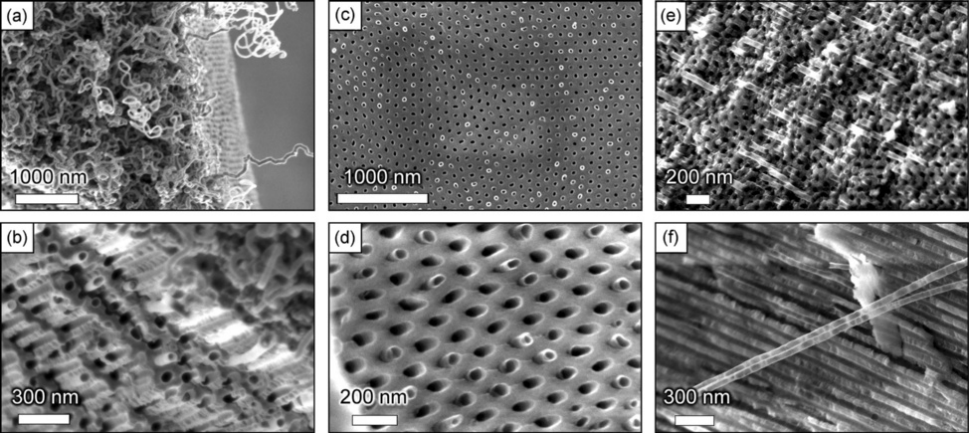
**SEM images. (a, b)** SEM images of the carbon nanotubes grown in the 750°C process, Fe + S1813 series (C_2_H_4_ + S1813 + Fe, see Table 1). Nanotubes protruding from the membrane channels are clearly visible in **(b)**. **(c, d)** SEM images of the carbon nanotubes grown in the 750°C process, Fe + S1813 + Plasma series (C_2_H_4_ + S1813 + plasma). **(e, f)** Nanotubes grown in the ‘900°C’ process, Fe + S1813 + Plasma series (CH_4_ + S1813 + plasma).

The experiments of the Fe + S1813 series, i.e. growth on samples prepared with the use of both Fe catalyst and S1813 photoresist, have demonstrated nucleation of the carbon nanotubes inside the membrane pores as well as the formation of a nanotube mat on the top of membrane, as can be seen in Figure 5a,b. Indeed, Figure 5a shows a dense nanotube layer on the membrane top, whereas Figure 5b which is an SEM image of the broken side surface of the membrane clearly reveals the origin of the nanotubes in the channels. Short ends of the nanotubes of about 100 to 200 nm are protruding from the channels of the membrane. More SEM images of the nanotubes grown in C_2_H_4_ with S1813 photoresist can be found in Additional file 1: Figure S2.

A better degree of control was obtained in Fe + S1813 + Plasma series, i.e. in growing the nanotubes on alumina plasma-treated membranes. Figure 5c,d shows SEM images of the nanotubes grown by 750°C process (C_2_H_4_ + S1813 + plasma). Importantly, the thick fibrous mat of entangled nanotubes was not found in this case, but all nanotubes look like they have been cut near the membrane surface. Moreover, the nanotube ends are not deformed, and the nanotubes are open. A similar experiment in CH_4_ (S1813 + Fe + plasma, at 900°C) has demonstrated a similar structure with many nanotubes protruding from the pores but not forming the mat (Figure 5e). Examination of the dissected membrane has revealed the presence of thin, straight carbon nanotubes within the channels (Figure 5f). More SEM images of the nanotubes grown on plasma-treated membranes can be found in Additional file 1: Figure S3.

It should be noted that SEM and TEM examinations reveal the open-end carbon nanotubes grown inside the channels and on the membrane top (see Figures 1, 4 and 5 in Additional file 1: Figures S2 and S3). Examination of many SEM images made at different tilt angles shows that most of the nanotubes have open ends. This important finding could be explained by the specific mechanism of the nanotube nucleation and growth on the nanoporous membranes. We believe that the surface features of the membrane surface play a major role in nanotube nucleation and sustaining the growth (a similar mechanism was described for the silicon surface with mechanically written features [32]). In this particular case, channel walls nucleate open nanotubes and sustain their growth with open ends. It should be also noted that the diameter of the channel-nucleated and grown nanotubes corresponds to the channel diameters (20 to 50 nm, Figure 5), whereas the diameters of the nanotubes nucleated on the membrane top can reach 70 to 80 nm (Figure 4). The number of atomic carbon layers composing the nanotube walls is also larger for the case of nanotubes nucleated on the membrane top.

Thus, the plasma posttreatment of the alumina membranes before the nanotube growth radically changes the outcomes. Indeed, nucleation of the nanotubes inside long channels becomes possible. Here, we should stress that we did not use any special catalyst applied into the channels (directly at the bottom), as it was demonstrated by other authors [33]. In contrast, we used a rather simple technique of depositing cheap and commonly used S1813 photoresist and a thin Fe layer onto the upper surface of the membrane. Most probably, the plasma posttreatment changes the energy state of the alumina membrane and promotes deep penetration of the photoresist (which serves as a carbon precursor) into the channels. As a result, nucleation and efficient growth of carbon nanotubes in the pores become possible.

To decide if the ion flux extracted from the plasma can penetrate into the channels in the alumina membrane and affect the surface state of the material, one should compare the thickness of the sheath between the plasma and the surface with the diameter of a typical channel (i.e. of about 50 nm) and estimate the typical ion energy colliding with the surface. For a floating surface, the surface potential *U*_S_ can be estimated [18,34]

(1) $$\lambda_{S}=\gamma_{S}{\left(\frac{\epsilon_{0}T_{e}}{en_{p}}\right)}^{\frac{1}{2}}U_{S}=\frac{kT_{e}}{e}\ln\left(\sqrt{\frac{2\pi m_{e}}{M_{i}}}\right),$$

where *T*_e_ is the electron temperature, *k* is the Boltzmann's constant, *e* is the electron charge, *m*_e_ is the electron mass and *M*_i_ is the ion mass. For typical low-temperature plasma parameters (*T*_e_ *≈* 2 to 3 eV), the surface potential is *U*_s_ *=* (5 to 7) × *T*_e_ = 10.20 eV. This energy is high enough as compared with the typical surface diffusion and re-evaporation energies (≈1 to 2 eV), and one can conclude that the ion flux is capable to change the surface state of the membrane and growth nanotubes. The sheath thickness for a typical plasma density (*n*_p_ ≈ 10^17^ to 10^18^ m^−3^) may be assumed to be of the order of a few Debye lengths [34]

(2) $$\lambda_{S}=\gamma_{S}{\left(\frac{\epsilon_{0}T_{e}}{en_{p}}\right)}^{\frac{1}{2}}\lambda_{S}=k_{p}\lambda_{D}=k_{p}{\left(\frac{\epsilon_{0}T_{e}}{en_{p}}\right)}^{\frac{1}{2}},$$

where *ϵ*_0_ is a dielectric constant, *λ*_D_ is the electron Debye length and *k*_p_ is the constant, typically in the range between 1 and 5. The estimates using Equation 2 give the sheath thickness of the order of 10 μm to 0.1 mm, that is, much larger than the average diameter of the alumina membrane channels. This means that the ions extracted from the plasma edge will not be significantly deflected by the electric field distorted by nanosized features on the membrane surface. Hence, the ions move along straight trajectories and could penetrate deeply into the channels. As a result, one can expect that the surface of the channels will be treated by the ion flux penetrating relatively deeply under the upper surface of the membrane.

**Figure 6:**
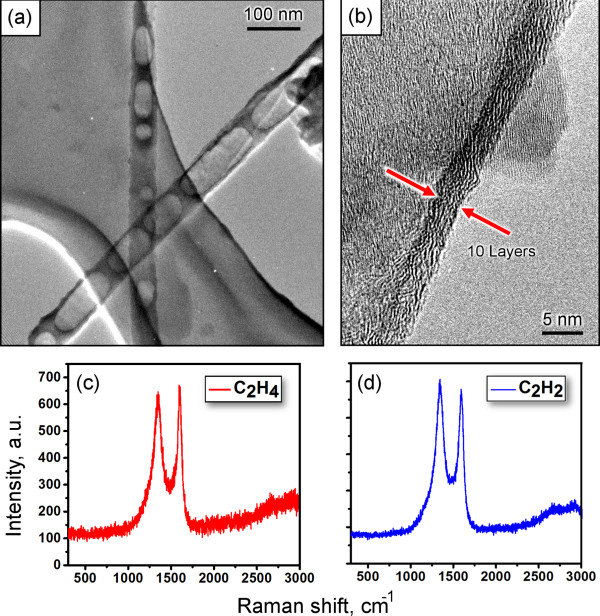
**TEM and Raman characterization. (a, b)** High-resolution TEM images of the carbon nanotubes grown on top of the alumina membrane with S1813 photoresist. A relatively thin wall consisting of 10 atomic carbon layers can be seen in **(b)**. **(c, d)** The Raman spectra of the nanotube grown using C_2_H_4_ and C_2_H_2_ precursors show D and G bands and a relatively low presence of defects.

The Raman spectra of the nanotubes grown using C_2_H_4_ and C_2_H_4_ precursors (Figure 6c,d) show D and G bands that are typical for multi-walled carbon nanotubes and a relatively low number of defects. The spectra of other samples are also very similar to those shown in Figure 6c,d, thus exhibiting relatively low defect level irrespective of the specific process conditions (see Additional file 1: Figure S5 for the Raman spectrum of nanotubes grown without S1813 photoresist). Further TEM analysis of the carbon nanotubes grown on top of alumina membrane with S1813 photoresist has demonstrated a rather good quality of the grown nanostructures with relatively thin walls consisting of approximately 10 atomic carbon layers (Figure 6a,b). More TEM images can be found in Additional file 1: Figures S4 and S6.

## Conclusions

To conclude, we have demonstrated that effective control of nucleation and growth of carbon nanotubes in nanopores of alumina membranes is possible by using plasma posttreatment of the membrane and application of S1813 photoresist as an additional carbon precursor. A few options to control the growth of nanotubes inside the membrane channels or on the upper membrane surface were considered and successfully demonstrated. In particular, we have demonstrated the fabrication of multi-walled carbon nanotubes on plasma-treated membranes. The nanotubes conformally filled the membrane channels and did not form mats on the membrane top. Thus, the growth mode can be controlled, and complex structures on the basis of nanotubes can be produced for various applications. A plausible nucleation and growth mechanism was also proposed on the basis of analysis of the plasma parameters. Further experiments with different types of plasmas are warranted to reveal the potential of this method for applications of organic-inorganic nanohybrid materials for energy storage, sensing and other emerging areas.

## Competing interests

The authors declare that they have no competing interests.

## Authors’ contributions

JF, IL and KO conceived the project. JF, ZH and SY performed the experiments. All authors analysed the data, discussed the results and contributed to the manuscript preparation. All authors read and approved the final manuscript.

## Supplementary Material

Additional file 1 **Temperature/time dependencies, three-dimensional visualization and SEM images.** Temperature/time dependencies for three processes used for growing carbon nanotubes on alumina membranes and three-dimensional visualization of the targeted structure and SEM images of the carbon nanotubes on AAO membrane. Click here for file
