# Supplementary material for: Carbon nanotubes on nanoporous alumina: from surface mats to conformal pore filling
Source: Nanoscale Res Lett. 2014 Aug 12;9(1):390. doi: 10.1186/1556-276X-9-390 (PMC4147107; doi:10.1186/1556-276X-9-390)
Supplement: Additional file 1 — Temperature/time dependencies, three-dimensional visualization and SEM images. Temperature/time dependencies for three processes used for growing carbon nanotubes on alumina membranes and three-dimensional visualization of the targeted structure and SEM images of the carbon nanotubes on AAO membrane. [file 1556-276X-9-390-S1.doc]

**Additional File 1**

**Carbon nanotubes on nanoporous alumina: from surface mats to conformal pore filling**

Jinghua Fang^1,2^, Igor Levchenko^1,3^, Zhao Jun Han^1^, Samuel Yick^1,3^, and Kostya (Ken) Ostrikov^1,3,4^

*^1^Plasma Nanoscience Laboratories, Manufacturing Flagship, CSIRO, P.O. Box 218, Lindfield, NSW 2070, Australia.*

*^2^ School of Physics, University of Melbourne, Parkville, VIC 3010, Australia.*

*^3^Complex Systems, School of Physics, The University of Sydney, Sydney, NSW 2006, Australia.*

*^4^Institute for Future Environments and School of Chemistry, Physics, and Mechanical Engineering, Queensland University of Technology, Brisbane, QLD 4000, Australia.*

*Correspondence and requests for materials should be addressed to I.L. (e-mail:* [*Igor.Levchenko@csiro.au*](mailto:Igor.Levchenko@csiro.au)*).*

**CONTENTS**

1. **Figure S1** Carbon nanotubes grown on alumina membrane in C_2_H_2_. The channels are empty, carbon nanotubes form a mat on the surface only.

2. **Figure S2** Carbon nanotubes grown on alumina membrane in C_2_H_4_+S1813 process. The nanotubes origin in the alumina channels and form a dense fibrous mat on the membrane top. Scale bars are 1 μm, 300 nm.

3. **Figure S3** Carbon nanotubes grown on alumina membrane in CH_4_+S1813 after the plasma treatment (Series 3, Process 1, 900 °C). The nanotubes origin in the alumina channels and DO NOT form a fibrous mat on the membrane top. Scale bars are 2 μm and 200 nm.

4. **Figure S4** TEM image of the carbon nanotubes grown on alumina membrane in CH_4_+S1813
after plasma treatment.

5. **Figure S5** Raman spectrum of the carbon nanotubes grown on alumina membrane without S1813

6. **Figure S6** TEM image of the carbon nanotubes grown on alumina membrane in CH_4_+S1813
after plasma treatment


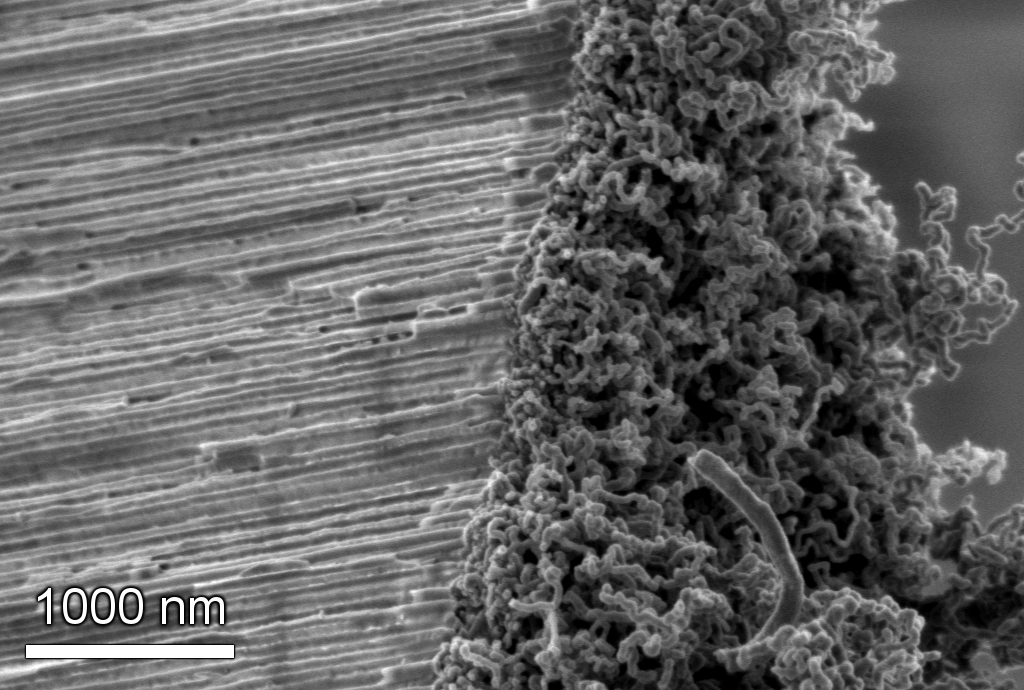


**Figure S1** Carbon nanotubes grown on alumina membrane in C_2_H_2_. The channels are empty, carbon nanotubes form a mat on the surface only.


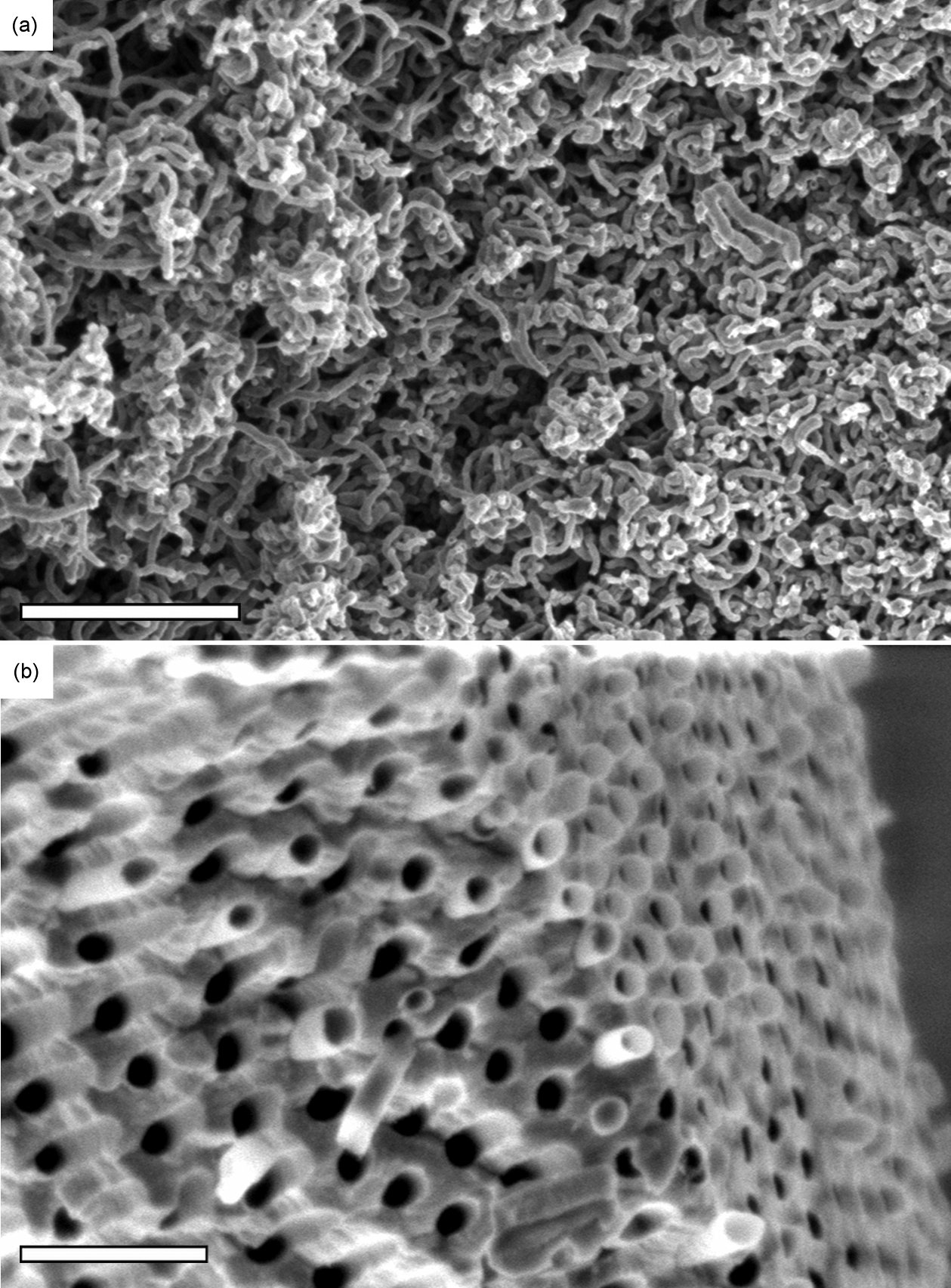


**Figure S2** Carbon nanotubes grown on alumina membrane in C_2_H_4_+S1813 process. The nanotubes origin in the alumina channels and form a dense fibrous mat on the membrane top. Scale bars are 1 μm, 300 nm.


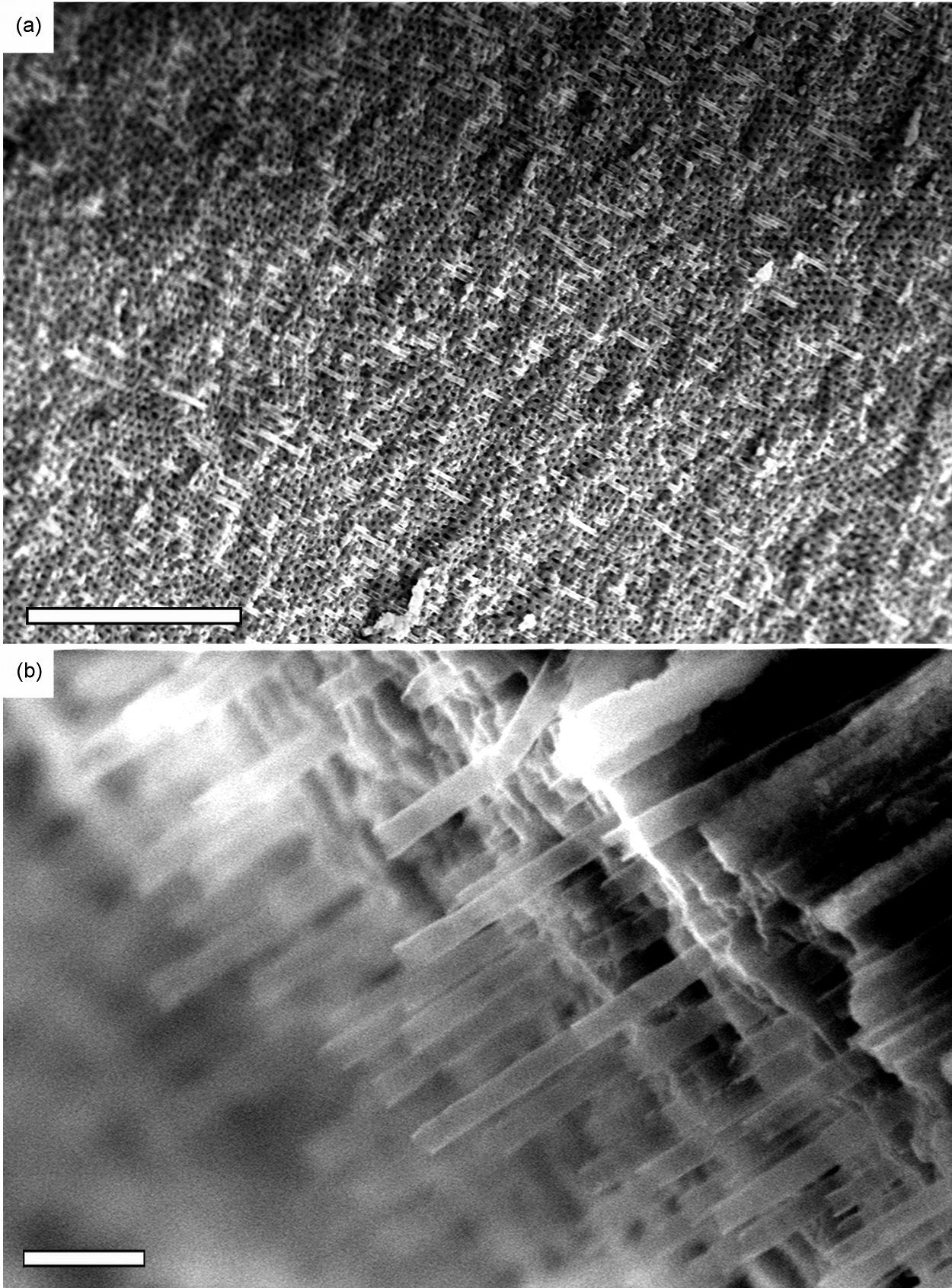


**Figure S3** Carbon nanotubes grown on alumina membrane in CH_4_+S1813 after the plasma treatment (Series 3, Process 1, 900 °C). The nanotubes origin in the alumina channels and DO NOT form a fibrous mat on the membrane top. Scale bars are 2 μm and 200 nm.


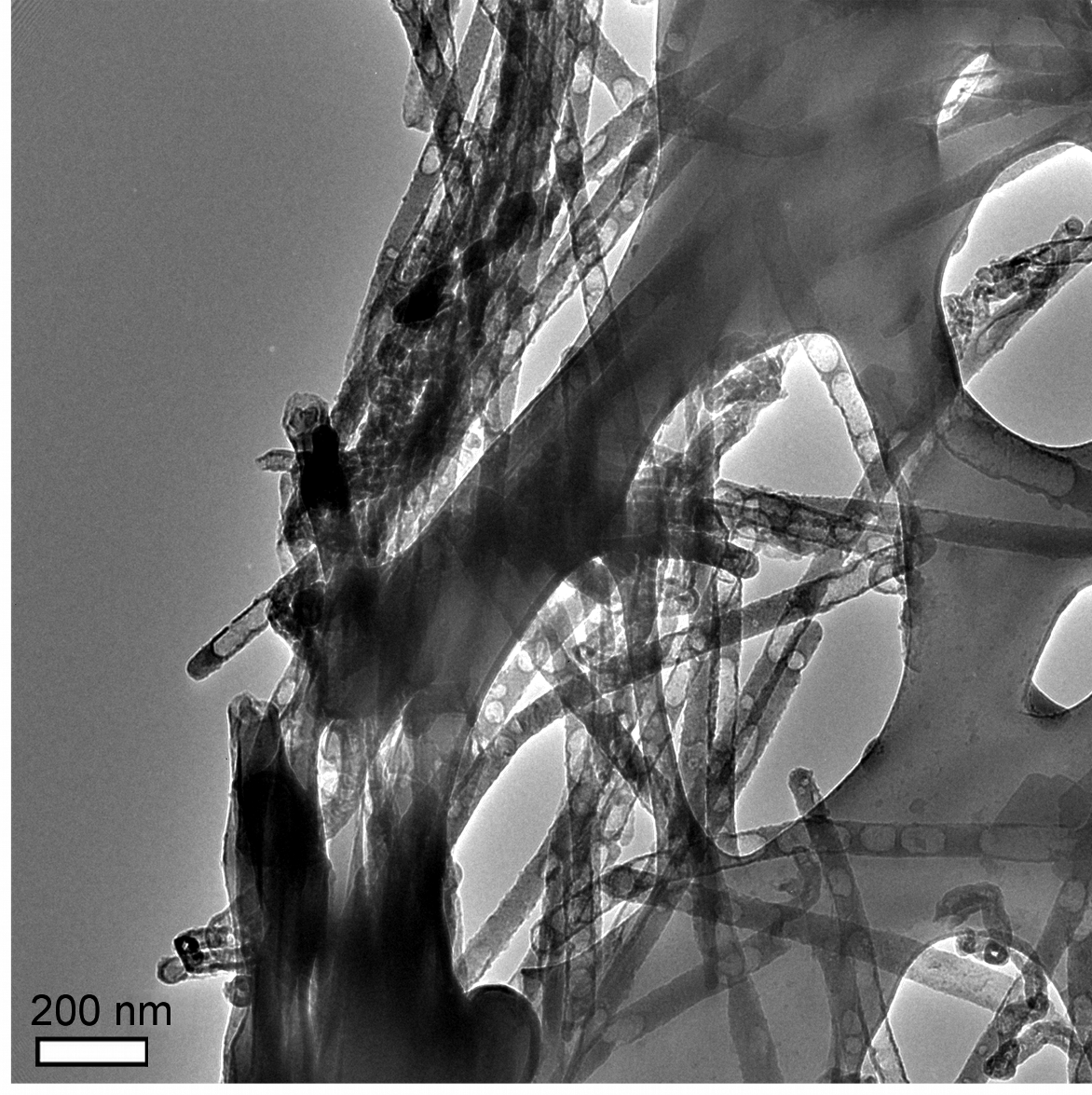


**Figure S4** TEM image of the carbon nanotubes grown on alumina membrane in CH_4_+S1813
after plasma treatment.


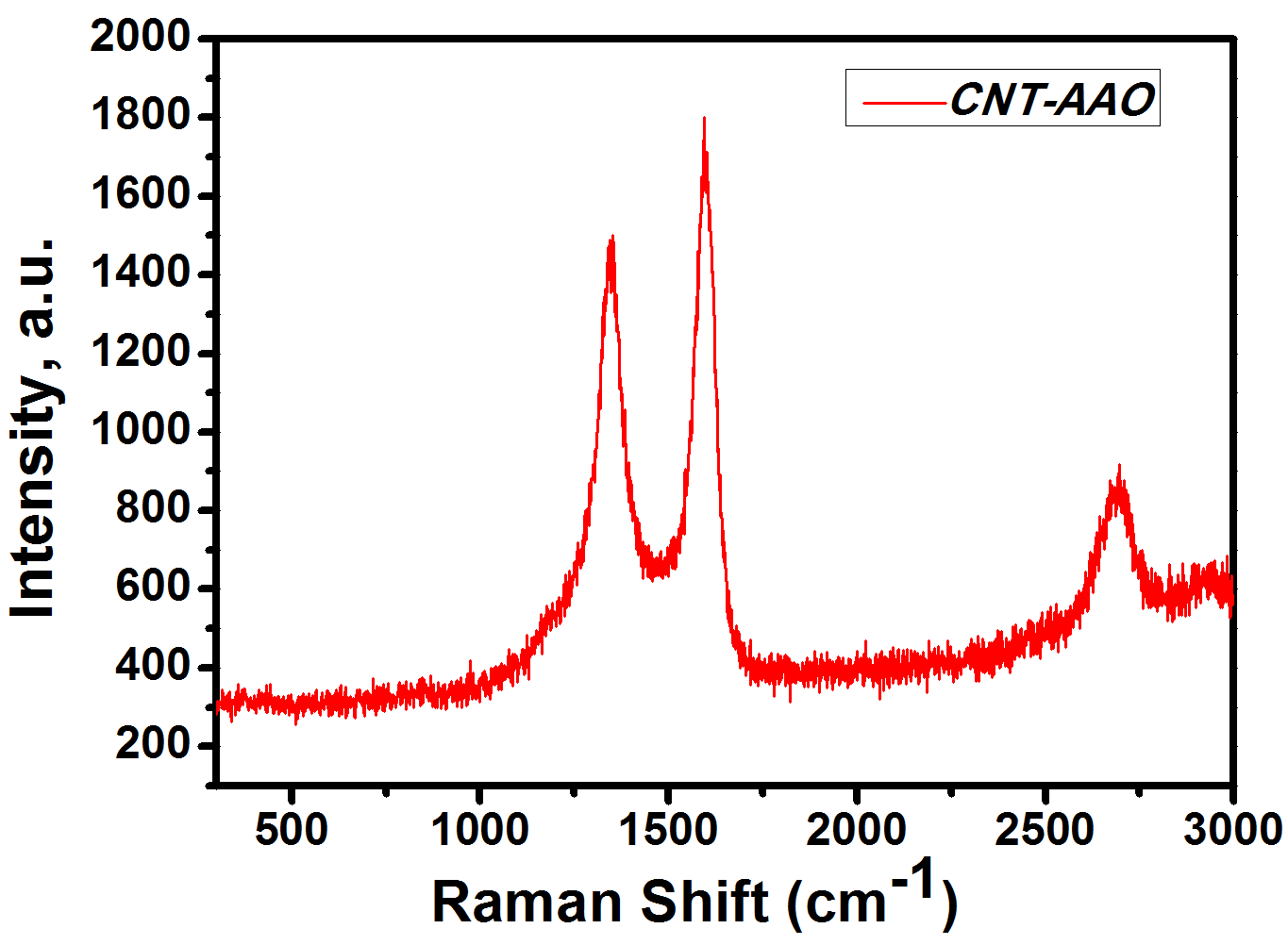


**Figure S5** Raman spectrum of the carbon nanotubes grown on alumina membrane without S1813.


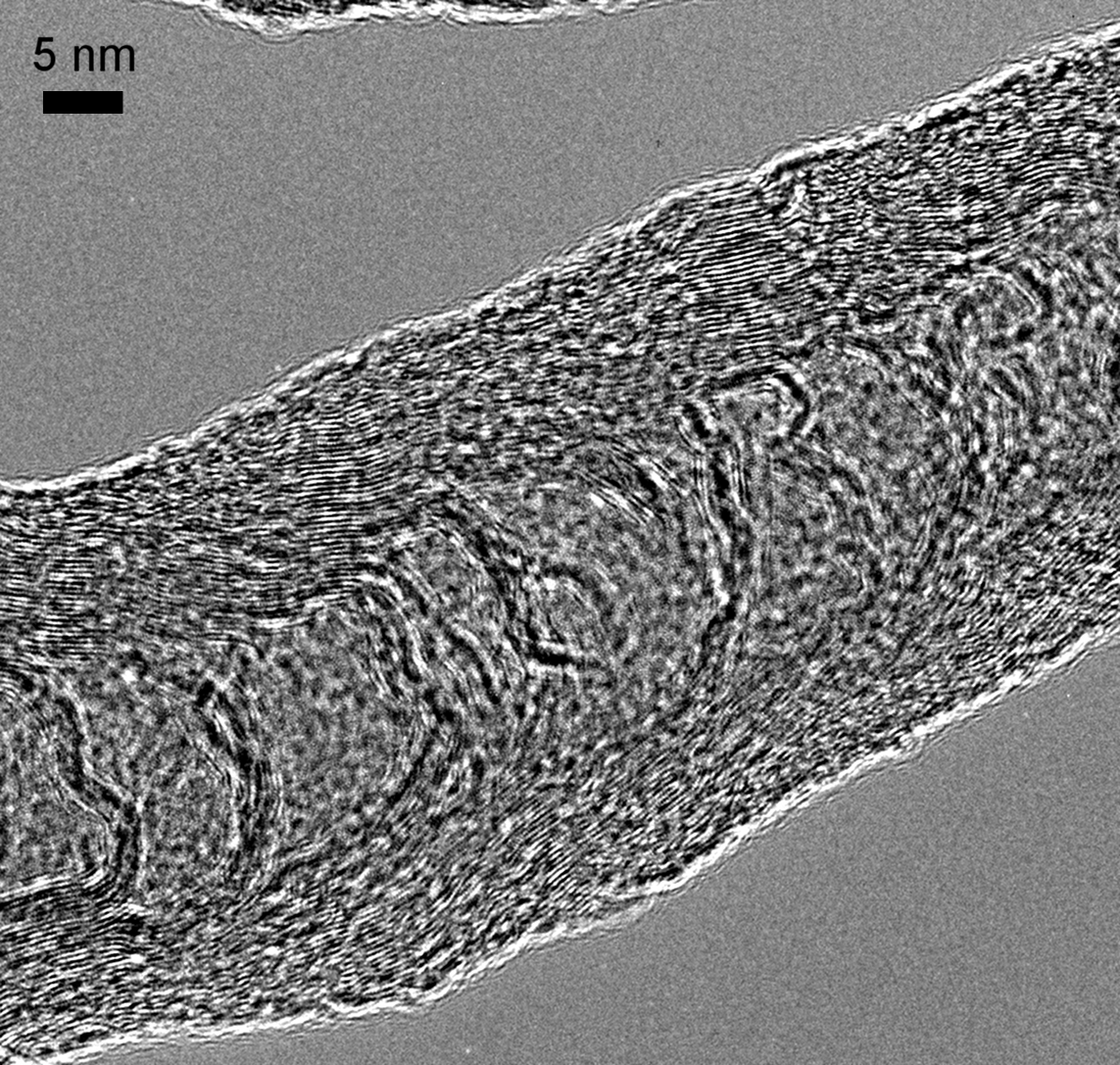


**Figure S6** TEM image of the carbon nanotubes grown on alumina membrane in CH_4_+S1813
after plasma treatment.
